# Plasma GFAP in Parkinson’s disease with cognitive impairment and its potential to predict conversion to dementia

**DOI:** 10.1038/s41531-023-00447-7

**Published:** 2023-02-09

**Authors:** Yilin Tang, Linlin Han, Shiyu Li, Tianyu Hu, Zhiheng Xu, Yun Fan, Xiaoniu Liang, Huan Yu, Jianjun Wu, Jian Wang

**Affiliations:** Department of Neurology and National Research Center for Aging and Medicine & National Center for Neurological Disorders, State Key Laboratory of Medical Neurobiology, Huashan Hospital, Fudan University, Shanghai, China

**Keywords:** Parkinson's disease, Predictive markers

## Abstract

Glial fibrillary acidic protein (GFAP) has been suggested as a biomarker for reactive astrogliosis. We measured the levels of plasma GFAP by Simoa in 60 patients with PD with normal cognition, 63 with mild cognitive impairment (PD-MCI), 24 with dementia (PDD) and 15 healthy controls. A subgroup of patients with PD-MCI (*n* = 31) was followed up for 4.1 ± 2.3 years. Compared with healthy controls, plasma GFAP levels were elevated in patients with PDD (adjusted *P* < 0.001) and PD-MCI (adjusted *P* = 0.013) and were negatively correlated with the Mini Mental State Examination (MMSE) score in PD participants. Plasma GFAP predicted MCI-to-dementia conversion with an AUC of 0.90, higher than NfL, Tau and pTau181. Our results support that plasma GFAP has potential value for distinguishing patients with PDD, and predicting MCI-to-dementia conversion in PD.

## Introduction

Parkinson’s disease (PD) is a neurodegenerative disease with complex molecular and clinical characteristics^1^. Cognitive impairment, including mild cognitive impairment (PD-MCI) and dementia (PDD), is a prevalent non-motor symptom of PD^1,2^.

Growing evidence indicates that neuroinflammatory processes such as reactive astrogliosis contribute to PD development^3,4^. Glial fibrillary acidic protein (GFAP) is an intermediate filament protein that is primarily expressed in astrocytes of the central nervous system. When the nervous system is injured, astrocytes rapidly release GFAP into the bloodstream^5^. A few studies have examined serum GFAP in PD, finding elevated serum levels of GFAP compared to controls^6,7^. However, there is a lack of studies investigating GFAP in PD from a cognitive perspective. Whether GFAP can be used as a potential biomarker to predict cognitive progression of PD is unknown. Here, we evaluated plasma GFAP levels to investigate differences in patients with PD with normal cognition (PD-NC), PD-MCI and PDD. We also investigated possible correlations between levels of GFAP and cognitive scores, as well as progression to dementia. Furthermore, we compared levels of plasma GFAP with three other neuronal blood markers, that is, neurofilament light chain (NfL), Tau and pTau181.

## Results

The demographic information of the study participants is presented in Table 1, and detailed observations of cognitive performance are reported in Supplementary Table 1. Increased Movement Disorders Society Unified Parkinson’s Disease Rating Scale part III (MDS UPDRS-III) scores were observed in the PDD group compared to the PD-NC group (adjusted *P* < 0.05). As expected, performance on the Mini Mental State Examination (MMSE) was worse in the PDD group as compared to age-matched healthy control subjects (HCs), PD-NC and PD-MCI groups (all adjusted *P* < 0.001).

**Table 1:** Demographic and clinical characteristics of the study groups.

	HCs	PD-NC	PD-MCI	PDD	*P* Values
Number of subjects	15	60	63	24	—
Age, years	61.93 (8.56)	58.45 (8.94)	59.60 (10.53)	63.83 (10.61)	0.124^a^
Sex (men/women)	6/9	31/29	41/22	14/10	0.245^b^
Education, years	10.00 (9.00, 12.00)	12.00 (9.00, 15.00)	9.00 (7.50, 12.50)	9.00 (7.75, 13.25)	0.177^c^
Disease duration, months	—	18.00 (9.75, 29.25)	13.00 (8.00, 24.00)	18.50 (10.50, 38.75)	0.388^c^
MDS UPDRS-III score	—	18.50 (14.75, 29.25)	24.00 (17.00, 38.00)^d^	30.00 (18.00, 49.00)^e^	0.019^c^
Hoehn and Yahr stage (1/2/3)	—	16/38/6	12/45/6	3/16/5	0.369^b^
LED, mg/day	—	150.00 (0.00, 356.25)^d^	150.00 (0.00, 300.00)	250.00 (0.00, 475.00)^d^	0.064^c^
BDI score	—	7.50 (2.00, 13.00)	12.00 (5.00, 19.00)^d^	15.00 (5.25, 24.75)^d^	0.021^c^
MMSE score	28.00 (27.00, 29.00)	29.00 (27.00, 29.00)	27.00 (26.00, 28.50)	22.50 (14.00, 24.00)^f,g,h^	<0.001^c^
Plasma GFAP (pg/ml)	71.99 (47.29, 95.90)	87.27 (66.64, 113.57)	93.07 (75.70, 125.71)^i^	145.79 (95.57, 214.30)^f,g,h^	<0.001^j^
Plasma NfL (pg/ml)	7.70 (5.35, 9.03)	11.42 (8.66, 19.10)^i^	12.98 (10.45, 17.32)^k^	21.61 (11.40, 28.47)^f,l,m^	0.001^j^
Plasma Tau (pg/ml)	3.35 (2.87, 3.94)	3.47 (2.54, 4.25)	3.47 (2.51, 4.28)	3.08 (1.96, 4.30)	0.451^j^
Plasma pTau181 (pg/ml)	1.33 (1.20, 1.73)	1.38 (0.96, 1.84)	1.54 (1.16, 2.09)	2.11 (1.54, 2.53)^i,l,m^	0.015^j^

Data are presented as the mean (SD) or median (25–75% quartile).

^a^one-way ANOVA followed by Bonferroni’s post hoc correction for multiple comparisons.

^b^*χ*^2^ test.

^c^Kruskal‒Wallis test with Dunn’s post hoc test.

^d^Missing values: MDS UPDRS-III score: PD-MCI 1; LED: PD-NC 1, PDD 1; BDI score: PD-MCI 2, PDD 1.

^e^*P* < 0.05 vs. PD-NC.

^f^*P* < 0.001 vs. HCs.

^g^*P* < 0.001 vs. PD-NC.

^h^*P* < 0.001 vs. PD-MCI.

^i^*P* < 0.01 vs. HCs.

^j^Generalized linear model adjusting for age.

^k^*P* < 0.05 vs. HCs.

^l^*P* < 0.01 vs. PD-NC.

^m^*P* < 0.05 vs. PD-MCI.

*HCs* healthy controls, *PD-NC* Parkinson’s disease with normal cognition, *PD-MCI* Parkinson’s disease with mild cognitive impairment, *PDD* Parkinson’s disease dementia, *MDS-UPDRS* Movement Disorders Society Unified Parkinson’s Disease Rating Scale, *LED* Levodopa equivalent dose, *BDI* Beck Depression Inventory, *MMSE* Minimum Mental State Examination, *GFAP* glial fibrillary acidic protein, *NfL* neurofilament light chain.

Age was significantly correlated with plasma GFAP (*r* = 0.158, *P* = 0.044) in all participants. We observed no significant correlation between plasma GFAP and disease duration (*r* = 0.019, *P* = 0.816), Hoehn and Yahr (H&Y) stage (*r* = 0.160, *P* = 0.063), or levodopa equivalent dose (LED, *r* = 0.049, *P* = 0.558) in all participants. Furthermore, in all participants, sex did not significantly affect plasma GFAP levels (Mann–Whitney U = 2899, *P* = 0.278). For these reasons, we included age as a covariate in the statistical comparison of the study groups.

**Fig. 1:**
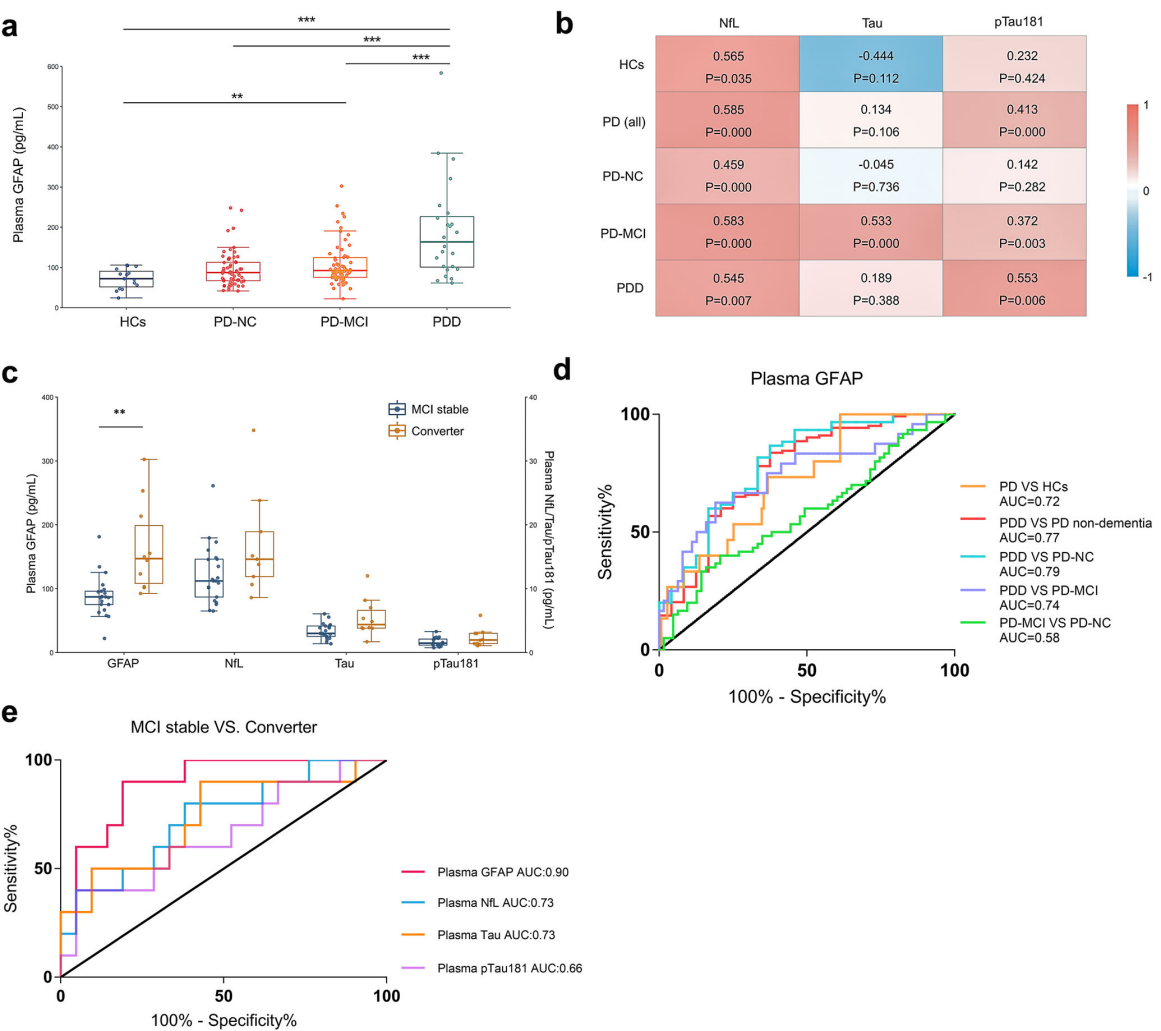
Diagnostic and prognostic performance of plasma GFAP. **a** Plasma GFAP levels in healthy controls (*n* = 15) and patients with PD-NC (*n* = 60), PD-MCI (*n* = 63) and PDD (*n* = 24). Groups were compared by a generalized linear model after adjusting for age. Boxes show the median and IQR, and whiskers are the minimum and maximum. Dots are single values. **b** Correlation analysis of plasma GFAP with plasma NfL, Tau and pTau181 using Spearman’s partial correlation including age as a covariate. The correlation coefficient r is given in the heat map. **c** Baseline levels of plasma GFAP, NfL, Tau and pTau181 in PD patients with MCI stable (*n* = 21) and conversion to dementia (converter, *n* = 10) during the whole follow-up period. Groups were compared by the Mann–Whitney *U* test. Boxes show the median and IQR, and whiskers are the minimum and maximum. Dots are single values. **d** Receiver operating characteristic (ROC) curve analysis indexed the efficiency of plasma GFAP for the differential diagnosis of Parkinson’s disease subgroups stratified according to cognitive status. **e** ROC curve analysis for baseline levels of plasma GFAP, NfL, Tau, and pTau181 to predict MCI-to-dementia conversion during the next 4.1 ± 2.3 years. *p < 0.05, **p < 0.01, ***p < 0.001. HCs healthy controls, PD-NC Parkinson’s disease with normal cognition, PD-MCI Parkinson’s disease with mild cognitive impairment, PDD Parkinson’s disease dementia, AUC area under the curve.

After adjusting for age, we found that plasma GFAP was significantly elevated in the PDD group compared to the HCs (adjusted *P* < 0.001), PD-NC (adjusted *P* < 0.001) and PD-MCI (adjusted *P* < 0.001) groups. Moreover, plasma GFAP was significantly increased in the PD-MCI group compared to the HCs (adjusted *P* = 0.009, Fig. 1a).

To evaluate whether plasma GFAP was correlated with performance on the MMSE, we used Spearman’s partial correlation including age as a covariate. A significant correlation between plasma GFAP and MMSE score was detected in all participants with PD (*r* = −0.584, *P* < 0.001). Furthermore, significant correlations between plasma GFAP and MMSE scores were detected in the PD-MCI (*r* = −0.313, *P* = 0.013) and PDD groups (*r* = −0.506, *P* = 0.014), but not observed in the PD-NC group (*r* = −0.128, *P* = 0.335). The correlations between GFAP and other neuropsychological tests are shown in Supplementary Table 2.

Next, we studied the relationship between plasma GFAP and three other plasma biomarkers, NfL, Tau and pTau181. Plasma GFAP showed a significant moderate correlation with plasma NfL (*r* = 0.585, *P* < 0.001) and pTau181 (*r* = 0.413, *P* < 0.001) in all PD participants (Fig. 1b).

A subgroup of patients with PD-MCI (*n* = 31) was clinically followed up for 4.1 ± 2.3 years. Twenty-one PD subjects showed stable MCI during the whole follow-up period whereas 10 PD patients with MCI progressed to dementia (converter). Plasma GFAP at baseline was significantly higher in converters than in patients with stable MCI (*P* < 0.01). None of the other three biomarkers (NfL, Tau and pTau181) showed significant differences between stable MCI and converters (Supplementary Table 3 and Fig. 1c).

ROC curve analysis revealed that plasma GFAP allowed a reliable differential diagnosis between PDD and PD-NC (AUC = 0.79), PD-MCI (AUC = 0.74) or PD with non-dementia (including both PD-NC and PD-MCI, AUC = 0.77) (Fig. 1d). Plasma GFAP was unsuitable to discriminate PD-MCI and PD-NC.

Furthermore, we investigated the predictive value of plasma GFAP, NfL, Tau and pTau181 for MCI to dementia conversion in PD. Plasma GFAP at baseline had a high accuracy for separating patients with stable MCI and MCI to dementia conversion (AUC = 0.90) after an average follow-up of 4.1 ± 2.3 years, with a sensitivity of 90% and specificity of 81% at an optimal cut-off of 100.2 pg/mL. The other three biomarkers showed lower performance in discriminating the two groups of patients (Fig. 1e).

## Discussion

In the present study, we found increased plasma GFAP levels in PD patients with dementia as well as MCI, compared to controls. Plasma GFAP showed a significantly negative correlation with the MMSE score in the PD participants. Furthermore, we suggest that plasma GFAP may be a potential biomarker for predicting the progression of MCI to dementia in PD.

Previous studies have demonstrated the presence of reactive astrogliosis in PD progression, but their role in the pathophysiology is not fully understood^3,4^. GFAP is a major structural component of fibrillary astrocytes and has proven to be one of the most common reactive astrocytic markers^5^. In this study, we found that levels of plasma GFAP increased in patients with PDD, and the increase was also observed in patients with PD-MCI. Plasma GFAP was negatively correlated with MMSE scores in the PDD and PD-MCI groups; however, no significant correlation was observed in the PD-NC group. Our study might indicate the presence of reactive astrogliosis in the early phase of cognitive impairment in PD.

Importantly, the capacity to predict the timeframe of disease progression is a critical issue in clinical practice and clinical trials. We next investigated whether plasma GFAP could predict more rapid cognitive decline in PD. In the subgroup of patients with PD-MCI who were clinically followed up for an average of 4.1 years, baseline plasma GFAP was significantly higher in patients who developed dementia than in those who remained stable with MCI. Meanwhile, the other three biomarkers, NfL, Tau and pTau181, which are related to neuronal injury and neurodegeneration, did not differ significantly between stable MCI and converters. With an AUC of 0.90, plasma GFAP levels separate patients with stable MCI and converters. Although the results need to be confirmed with a larger sample size, it is worth noting that plasma GFAP measurement may be useful as a marker for predicting the transition from MCI to dementia in PD. This might be helpful for patient classification in clinical trials or as a follow-up marker.

This study has several limitations. First, our study used clinical diagnosis in the absence of neuropathological confirmation. However, patients were well characterized, and most of the patients were followed over time to acquire as accurate a clinical diagnosis as possible. Second, the number of participants in the study is limited. The results therefore need to be reproduced in larger cohorts. Finally, MMSE scores exhibit ceiling/floor effects. More sensitive scales of global cognitive abilities would need to be applied to further investigate the relationship of GFAP with cognitive function.

In conclusion, we demonstrate that the levels of plasma GFAP are elevated in PD patients with cognitive impairment, but not in PD patients with normal cognition. We suggest that plasma GFAP might have prognostic value for predicting MCI-to-dementia conversion in PD.

## Methods

### Participants and controls

The study participants were recruited from the Movement Disorders Clinics at Huashan Hospital, Fudan University, between April 2013 and October 2021. Patients with PD-NC (*n* = 60), PD-MCI (*n* = 63), PDD (*n* = 24), and HCs (*n* = 15) were enrolled. PD subjects were diagnosed according to the UK Brain Bank criteria^8^. All HCs had a negative history of neurological or psychiatric disorders. A subgroup of patients with PD-MCI (*n* = 31) was clinically followed up for 4.1 ± 2.3 years (mean ± SD) after sample collection (baseline), and the details are presented in Supplementary Fig. 1 and Supplementary Table 4. The clinical and neuropsychological features were assessed annually. Ethics approval for the study was received from the Institutional Review Board at Huashan Hospital (approval numbers: 2011-174-2). All participants provided written informed consent in accordance with the Declaration of Helsinki.

### Clinical and neuropsychological assessments

Patients underwent clinical assessment at least 12 h off anti-parkinsonian medications. Disease severity was evaluated using the MDS UPDRS-III and the H&Y scale^9^. The Beck Depression Inventory (BDI) was performed to evaluate depression^10^. The dosage of antiparkinsonian drugs was converted into a total daily LED^11^.

The MMSE was performed to assess global cognitive function^12^. A full set of neuropsychological tests for five specific cognitive domains were carried out as follows: (1) Symbol Digit Modality Test (SDMT) and Trail Making Test A (TMT-A) for attention and working memory^13,14^, (2) Trail Making Test B (TMT-B) and Stroop Color-Word Test (CWT) for executive function^15,16^, (3) Animal Fluency Test (AFT) and Boston Naming Test (BNT) for language^16^, (4) Auditory Verbal Learning Test (AVLT) and Rey-Osterrieth Complex Figure Test (CFT-delay) for memory^17,18^, (5) Rey-Osterrieth Complex Figure Test (CFT-copy) and Clock Drawing Test (CDT) for visuospatial function^18,19^.

PDD was diagnosed according to the MDS criteria^20^. The MDS Task Force Level 2 was applied for PD-MCI diagnosis^21^. The PD patients who were excluded from PD-MCI and PDD were defined as PD-NC.

### Plasma sample collection and storage

After a 12 h overnight fast, blood samples were collected in the morning in EDTA Vacutainer tubes (BD). EDTA blood were briefly stored on ice and then was centrifuged at 2000 × *g* for 15 min within 30 min after collection. Plasma supernatant was collected, divided into aliquots (200 µl/tube), and frozen at − 80 °C until further use.

### Measurement of GFAP, NfL, Tau, and p-Tau181 in plasma

Plasma EDTA samples were thawed and centrifuged at 10,000 × *g* for 5 min at room temperature. Plasma GFAP, NfL, and Tau were measured using the HD-X Neurology 4-Plex B Kit, and plasma pTau181 was detected using the HD-X Simoa pTau181 V2 Advantage Kit. These two kits were all 2-step digital immunoassays. Assay calibrants provided in the kit were added in 96-well plates together with samples to make standard curves. Beads were vortex for 30 s immediately before loading reagents (Bead Reagent, Sample Diluent, Detector Reagent, and SBG Reagent). Samples were detected with the Quanterix Simoa HD-X analyzer using the standard 4x Dilution setup, and assay calibrants were run neat in triplicates. The concentration of GFAP, NfL, Tau, and pTau181 in unknown samples is interpolated from corresponding standard curve obtained by logistical regression fittings. GFAP, NfL, and Tau measurements of all patients were performed on the same day, and so did the pTau181 analysis. Samples were randomized, blinded, and measured using a batch of reagents from the same lot. All assays showed good analytical performance. The within-run variations and between-run variations ranged consistently below 15%.

### Statistical analysis

Categorical variables, including sex and H&Y stage, were assessed by performing the chi-square test. Demographic parameters between groups were evaluated using one-way ANOVA followed by Bonferroni’s corrected post hoc comparisons (for parametric data) and or Kruskal‒Wallis test followed by Dunn’s corrected post hoc comparisons (for non-parametric data). Correlation between plasma GFAP and demographic information, including age, disease duration, H&Y stage and LED, was performed with Spearman’s correlation analysis. The generalized linear model (GLM) was used to compare the levels of plasma GFAP, NfL, Tau, and pTau181 between groups after adjusting for age. Correlation analysis of plasma GFAP with MMSE score and other biomarkers using Spearman’s partial correlation including age as a covariate. Baseline levels of plasma GFAP, NfL, Tau, and pTau181 in patients with stable MCI and MCI-to-dementia converters were compared by the Mann–Whitney *U* test. The diagnostic performance of biomarkers was compared using receiver operating characteristic (ROC) curve analysis and cut-offs calculated by maximizing the Youden index. Statistical analysis was performed with IBM SPSS Statistics (version 26.0) and R (version 4.1.2). A *p* value < 0.05 was regarded as significant.

### Reporting summary

Further information on research design is available in the Nature Research Reporting Summary linked to this article.

## Supplementary information

Supplementary Materials

Reporting Summary

## Data Availability

The data used for this work are available from the corresponding authors upon reasonable requests. Restrictions may be applied to sensitive data for privacy preservation.
