# Supplementary material for: Plasma GFAP in Parkinson’s disease with cognitive impairment and its potential to predict conversion to dementia
Source: NPJ Parkinsons Dis. 2023 Feb 9;9:23. doi: 10.1038/s41531-023-00447-7 (PMC9911758; doi:10.1038/s41531-023-00447-7)
Supplement: Supplementary file 1 — Supplementary Materials [file 41531_2023_447_MOESM1_ESM.pdf]

Supplementary Materials

Supplementary Figure 1. Follow-up data of the patients with PD-MCI ...…………2

Supplementary Table 1. Detailed cognitive profile of the patients with Parkinson's disease….……………………………………………………………………...3-4

Supplementary Table 2. Correlations between plasma GFAP levels and neuropsychological tests in patients with Parkinson's disease….…………….……5-6

Supplementary Table 3. Baseline demographic and clinical characteristics of MCI stable and converter groups……….……………………..…………………….…7-8

Supplementary Table 4. Baseline demographic and clinical characteristics of patients with PD-MCI with follow-up and those without follow-up..………………...9

Supplementary Figure 1. Follow-up data of the patients with PD-MCI.

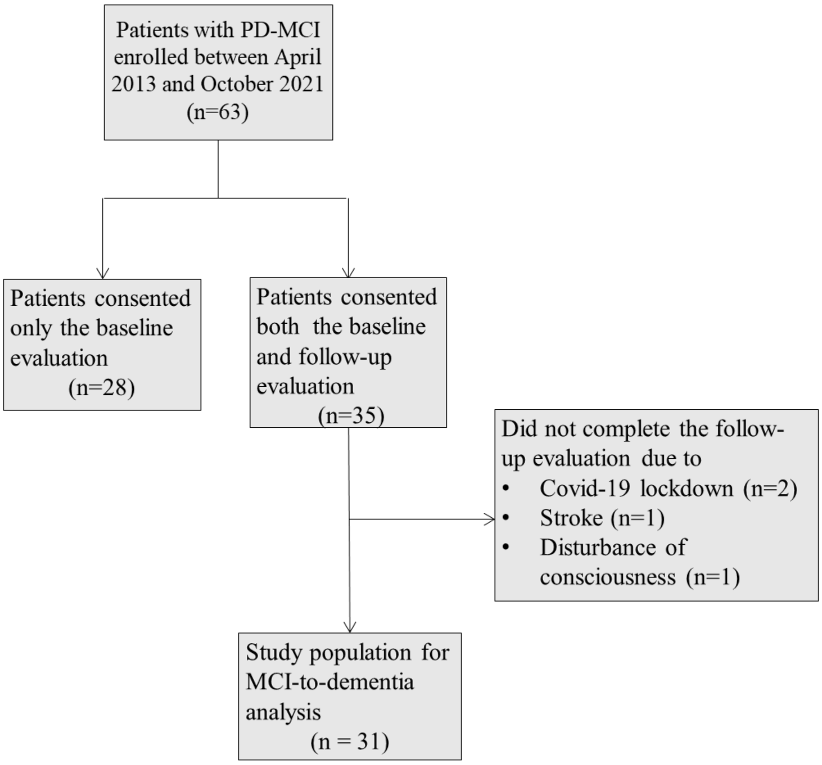

Follow-up visits	2	3	4	5	6	7	8	9
No. of cases	31	19	14	11	10	7	2	2
No. of cases with MCI stable	21	13	10	7	6	3	1	1
No. of cases with conversion	10	6	4	4	4	4	1	1

Supplementary Table 1. Detailed cognitive profile of the patients with Parkinson's disease

Cognitive test	PD-NC	PD-MCI	PDD	P Values[a]
**Attenion and working memory**				
SDMT	41.38 (14.52)	31.32 (14.73)	23.00 (19.60)	<0.001
TMT-A (s)	53.77 (13.05)	81.21 (39.02)	123.16 (93.72)	<0.001
**Executive function**				
CWT-C time (s)	76.25 (21.77)	96.50 (46.21)	123.24 (59.60)	<0.001
CWT-C right	46.53 (2.87)	44.23 (6.51)	36.11 (10.73)	<0.001
TMT-B (s)	127.48 (54.03)	199.18 (100.78)	192.19 (98.02)	<0.001
**Language**				
BNT	23.46 (3.81)	21.27 (4.44)	16.04 (5.39)	<0.001
AFT	16.86 (5.64)	14.49 (4.64)	9.63 (3.71)	<0.001
**Memory**				
AVLT-delay recall	4.82 (2.74)	2.87 (2.25)	1.72 (1.93)	<0.001
AVLT-T	25.43 (9.47)	19.48 (8.47)	15.33 (8.00)	<0.001
CFT-delay recall	15.07 (7.56)	10.55 (6.96)	7.39 (4.73)	<0.001
**Visuospatial function**				
CFT	33.93 (8.97)	31.03 (29.72)	20.10 (13.18)	0.039
CDT	23.19 (4.98)	18.68 (7.66)	12.57 (9.14)	<0.001

Data are presented as mean (SD)

[a] one-way ANOVA followed by Bonferroni's post hoc correction for multiple comparisons

PD-NC, Parkinson's disease with normal cognition; PD-MCI, Parkinson's disease with mild cognitive impairment; PDD, Parkinson's disease dementia; SDMT, Symbol Digit Modality Test; TMT, Trail Making Test; CWT, Stroop Color-Word Test; BNT,

Boston Naming Test; AFT, Animal Fluency Test; AVLT, Auditory Verbal Learning Test; CFT, the Rey-Osterrieth Complex Figure Test; CDT, Clock Drawing Test.

Supplementary Table 2. Correlations between plasma GFAP levels and neuropsychological tests in patients with Parkinson's disease.

**Cognitive test**	r	P Values
**Attention and working memory**		
SDMT	-0.104	0.222
TMT-A (s)	0.080	0.352
**Executive function**		
CWT-C time (s)	0.217	0.011
CWT-C right	-0.122	0.155
TMT-B (s)	0.291	0.001
**Language**		
BNT	-0.198	0.018
AFT	-0.118	0.167
**Memory**		
AVLT-delay recall	0.010	0.908
AVLT-T	0.003	0.969
CFT-delay recall	0.210	0.015
**Visuospatial function**		
CFT	-0.114	0.180
CDT	-0.155	0.154

Correlation analysis of GFAP with neuropsychological tests was performed using Spearman's partial correlation including age as a covariate.

Abbreviations: SDMT, Symbol Digit Modality Test; TMT, Trail Making Test; CWT, Stroop Color-Word Test; BNT, Boston Naming Test; AFT, Animal Fluency Test;

AVLT, Auditory Verbal Learning Test; CFT, the Rey-Osterrieth Complex Figure Test; CDT, Clock Drawing Test.

Supplementary Table 3. Baseline demographic and clinical characteristics of MCI stable and converter groups.

	MCI stable	Converter	P Values
**Number of subjects**	21	10	—
**Average follow-up years**	3.00 (2.00, 6.00)	3.00 (2.00, 7.00)	0.743
**Age, years**[a]	57.48 (10.98)	63.10 (9.06)	0.171
**Sex (men/women)**[b]	15/6	6/4	0.525
**Education, years**	12.00 (9.00, 16.00)	12.00 (10.00, 13.00)	0.262
**Disease duration, months**	14.00 (7.00, 24.00)	11.50 (8.50, 22.00)	0.800
**MDS UPDRS-III score**	23.00 (16.00, 31.00)	20.00 (17.00, 24.50)	0.966
**Hoehn and Yahr stage (1/2/3)**[b]	3/16/2	2/7/1	0.917
**LED, mg/day**	100.00 (0.00, 300.00)	100.00 (0.00, 293.75)	0.653
**BDI score**	12.00 (5.00, 18.00)	11.50 (5.00, 21.25)	0.946
**MMSE score at baseline**	29.00 (27.00, 29.00)	28.50 (26.00, 29.00)	0.456
**MMSE score at last visit**	28.00 (27.00, 29.00)	23.50 (18.00, 26.5)	0.003
**Plasma GFAP (pg/ml)**	87.19 (75.23, 95.98)	152.63 (133.75, 233.45)	0.002
**Plasma NfL (pg/ml)**	11.22 (8.69, 14.62)	14.88 (12.85, 21.37)	0.185
**Plasma Tau (pg/ml)**	3.00 (2.53, 4.15)	5.10 (3.85, 7.60)	0.098
**Plasma pTau181 (pg/ml)**	1.48 (1.16, 2.11)	1.68 (1.32, 2.61)	0.369

Data are presented as mean (SD) or median (25%–75% quartile)

[a] Student's *t*-test

[b] Chi-square test.

The other variables were compared using the Mann-Whitney U test

MCI, mild cognitive impairment; MCI stable, PD patients with stable MCI during the whole follow-up period; Converter, PD patients with MCI progressed to dementia during the whole follow-up period; MDS-UPDRS, Movement Disorders Society Unified Parkinson's Disease Rating Scale; LED, Levodopa equivalent dose; BDI,

Beck Depression Inventory; MMSE, Minimum Mental State Examination; GFAP, glial fibrillary acidic protein; NfL, neurofilament light chain.

Supplementary Table 4. Baseline demographic and clinical characteristics of patients with PD-MCI with follow-up and those without follow-up.

	PD-MCI with follow-up	PD-MCI without follow-up	P Values
**Number of subjects**	31	32	—
**Age, years**[a]	59.91 (10.64)	59.29 (10.59)	0.819
**Sex (men/women)**[b]	21/10	20/12	0.663
**Education, years**	9.00 (8.75, 12.00)	12.00 (9.00, 14.50)	0.104
**Disease duration, months**	13.50 (10.00, 22.75)	11.00 (7.00, 24.00)	0.912
**MDS UPDRS-III score**	30.00 (16.75, 40.00)	24.00 (16.00, 31.50)	0.069
**Hoehn and Yahr stage (1/2/3)**[b]	5/23/3	7/22/3	0.844
**LED, mg/day**	112.50 (0.00, 300.00)	100.00 (0.00, 300.30)	0.737
**BDI score**	11.50 (5.00, 18.00)	12.00 (6.00, 19.50)	0.834
**MMSE score**	26.00 (25.00, 27.00)	27.00 (24.20, 28.00)	0.116
**Plasma GFAP (pg/ml)**	90.59 (75.70, 120.69)	87.70 (77.93, 114.600)	0.441
**Plasma NfL (pg/ml)**	13.44 (10.95, 22.94)	11.68 (8.40, 15.45)	0.611
**Plasma Tau (pg/ml)**	3.10 (2.41, 3.93)	3.89 (2.81, 4.42)	0.070
**Plasma pTau181 (pg/ml)**	1.66 (1.13, 2.04)	1.48 (1.15, 2.07)	0.221

Data are presented as mean (SD) or median (25%–75% quartile)

[a] Student's *t*-test

[b] Chi-square test

The other variables were compared using the Mann-Whitney U test.

MCI, mild cognitive impairment; MCI stable, PD patients with stable MCI during the whole follow-up period; Converter, PD patients with MCI progressed to dementia during the whole follow-up period; MDS-UPDRS, Movement Disorders Society Unified Parkinson's Disease Rating Scale; LED, Levodopa equivalent dose; BDI, Beck Depression Inventory; MMSE, Minimum Mental State Examination; GFAP, glial fibrillary acidic protein; NfL, neurofilament light chain.
