# Supplementary material for: Plasma GFAP in Parkinson’s disease with cognitive impairment and its potential to predict conversion to dementia
Source: NPJ Parkinsons Dis. 2023 Feb 9;9:23. doi: 10.1038/s41531-023-00447-7 (PMC9911758; doi:10.1038/s41531-023-00447-7)
Supplement: Supplementary file 2 — Reporting Summary [file 41531_2023_447_MOESM2_ESM.pdf]

nature portfolio

Corresponding author(s): Jian Wang

Last updated by author(s): Dec 24, 2022

Reporting Summary

Nature Portfolio wishes to improve the reproducibility of the work that we publish. This form provides structure for consistency and transparency in reporting. For further information on Nature Portfolio policies, see our Editorial Policies and the Editorial Policy Checklist.

Statistics

For all statistical analyses, confirm that the following items are present in the figure legend, table legend, main text, or Methods section.

n/a	Confirmed	
☐	☒	The exact sample size (*n*) for each experimental group/condition, given as a discrete number and unit of measurement
☐	☒	A statement on whether measurements were taken from distinct samples or whether the same sample was measured repeatedly
☐	☒	The statistical test(s) used AND whether they are one- or two-sided<br>*Only common tests should be described solely by name; describe more complex techniques in the Methods section.*
☐	☒	A description of all covariates tested
☐	☒	A description of any assumptions or corrections, such as tests of normality and adjustment for multiple comparisons
☐	☒	A full description of the statistical parameters including central tendency (e.g. means) or other basic estimates (e.g. regression coefficient) AND variation (e.g. standard deviation) or associated estimates of uncertainty (e.g. confidence intervals)
☐	☒	For null hypothesis testing, the test statistic (e.g. *F*, *t*, *r*) with confidence intervals, effect sizes, degrees of freedom and *P* value noted<br>*Give P values as exact values whenever suitable.*
☒	☐	For Bayesian analysis, information on the choice of priors and Markov chain Monte Carlo settings
☒	☐	For hierarchical and complex designs, identification of the appropriate level for tests and full reporting of outcomes
☐	☒	Estimates of effect sizes (e.g. Cohen's *d*, Pearson's *r*), indicating how they were calculated

Our web collection on statistics for biologists contains articles on many of the points above.

Software and code

Policy information about availability of computer code

Data collection	no software was used
Data analysis	The data analysis was conducted by IBM SPSS Statistics (version 26.0) and R (version 4.1.2)

For manuscripts utilizing custom algorithms or software that are central to the research but not yet described in published literature, software must be made available to editors and reviewers. We strongly encourage code deposition in a community repository (e.g. GitHub). See the Nature Portfolio guidelines for submitting code & software for further information.

Data

Policy information about availability of data

All manuscripts must include a data availability statement. This statement should provide the following information, where applicable:

- Accession codes, unique identifiers, or web links for publicly available datasets
- A description of any restrictions on data availability
- For clinical datasets or third party data, please ensure that the statement adheres to our policy

We can share our relevant raw data supporting our findings. If any scientist wish to use them for non-commercial purposes, without breaching participant confidentiality, he/her can contact us directly, and we will share our raw data freely with he/her.

Human research participants

Policy information about studies involving human research participants and Sex and Gender in Research.

Reporting on sex and gender	In Chinese culture, sex (biological attribute) and gender (shaped by social and cultural circumstances) were consistent, so the disaggregated sex and gender data wasn't collected. Sex- and gender-based analyses weren't performed. Sex was determined based on biological concept, and our findings applied to sex.
Population characteristics	See below.
Recruitment	Patients were recruited from the Movement Disorders Clinics at Huashan Hospital, Fudan University. PD subjects were diagnosed according to the UK Brain Bank criteria. All health controls had a negative history of neurological or psychiatric disorders. The clinical and neuropsychological features were assessed annually. All participants provided written informed consent in accordance with the Declaration of Helsinki.
Ethics oversight	This study approved by the Human Studies Institutional Review Board, Huashan Hospital, Fudan University.

Note that full information on the approval of the study protocol must also be provided in the manuscript.

Field-specific reporting

Please select the one below that is the best fit for your research. If you are not sure, read the appropriate sections before making your selection.

☐ Life sciences ☒ Behavioural & social sciences ☐ Ecological, evolutionary & environmental sciences

For a reference copy of the document with all sections, see nature.com/documents/nr-reporting-summary-flat.pdf

Behavioural & social sciences study design

All studies must disclose on these points even when the disclosure is negative.

Study description	qualitative cohort study
Research sample	Participants were Chinese patients with Parkinson's disease who was diagnosed at Huashan Hospital, Fudan University. Demographic information included age, sex, disease duration, education, levodopa equivalent dose, Hoehn and Yahr stage, MMSE, BDI and MDS-UPDRS ?. The sample is not representative.
Sampling strategy	The sampling procedure was convenient. No sample-size calculation was performed. All participants conformed to the criteria were included in this study.
Data collection	Data of the participants were collected by pen and paper, for self-reported and evaluation of two senior investigators of movement disorders. The results were than updated to the database for storage. The researcher was blind during data collection.
Timing	The start and stop time of data collection was April 2013 and October 2021, separately. Patients were followed up annually in the same month (±2 weeks) in the previous year in same institution if available.
Data exclusions	32 patients with PD-MCI were excluded from the followed-up cohort and the exclusion reasons were shown in Supplementary Figure 1.
Non-participation	4 cases did not complete the follow-up evaluation (Covid-19 lockdown, n=2; stroke, n=1; disturbance of consciousness, n=1)
Randomization	Patients were allocated to groups based on PD diagnosis and cognition status.

Reporting for specific materials, systems and methods

We require information from authors about some types of materials, experimental systems and methods used in many studies. Here, indicate whether each material, system or method listed is relevant to your study. If you are not sure if a list item applies to your research, read the appropriate section before selecting a response.

Materials & experimental systems

n/a	Involved in the study
☒	☐ Antibodies
☒	☐ Eukaryotic cell lines
☒	☐ Palaeontology and archaeology
☒	☐ Animals and other organisms
☒	☐ Clinical data
☒	☐ Dual use research of concern

Methods

n/a	Involved in the study
☒	☐ ChIP-seq
☒	☐ Flow cytometry
☒	☐ MRI-based neuroimaging
